# Genomic basis of evolutionary change: evolving immunity

**DOI:** 10.3389/fgene.2015.00222

**Published:** 2015-06-19

**Authors:** Bregje Wertheim

**Affiliations:** Groningen Institute for Evolutionary Life Sciences, University of Groningen, Groningen, Netherlands

**Keywords:** gene interaction networks, adaptation, evolution, molecular mechanisms, complex traits, innate immunity, parasitoid resistance

## Abstract

Complex traits are manifestations of intricate gene interaction networks. Evolution of complex traits revolves around the genetic variation in such networks. Genomics has increased our ability to investigate the complex gene interaction networks, and characterize the extent of genetic variation in these networks. Immunity is a complex trait, for which the ecological drivers and molecular networks are fairly well understood in *Drosophila*. By characterizing the natural variation in immunity, and mapping how the genome changes during the evolution of immunity in *Drosophila*, we can integrate our knowledge on the complex genetic architecture of traits and the molecular basis of evolutionary processes.

## Introduction

Many traits are regulated and coordinated by multiple genes and environmental conditions. In nature, such traits evolve continuously under local selection pressures and neutral processes, leading to a rich diversity of phenotypic varieties and environmental coping strategies. Already, Dobzhansky (1964) described the rich diversity in the living world as the outcome of genetic diversity, environmental heterogeneity, and adaptations that evolve at the interplay between molecular and organismic biology. The molecular mechanisms in evolution have been particularly difficult to characterize. Firstly, we need to link the genotype to the phenotype. This link consists of extensive and intricate gene interaction networks (Ayroles et al., 2009; Lehner, 2013). Secondly, we need to identify the molecular changes responsible for phenotypic adaptations. This hinges on elucidating the genetic variation and the genetic changes that may occur anywhere in the gene interaction networks (e.g., Edwards et al., 2009).

The manifestation of genetic variation in gene networks is very complex. Changes in single genes may affect the activity and even topology of the whole genetic network (e.g., Knight et al., 2006). Genes and gene interaction networks are often pleiotropic and regulate various traits and processes, implying that changes in a single gene may be manifested in several traits (Stearns, 2010). Conversely, allelic variations in many genes may contribute to the variation in a particular phenotypic trait (Manolio et al., 2009). Furthermore, epistasis is pervasive, implying that allelic variations at multiple loci may affect the phenotypic effects of each other (Phillips, 2008; Mackay and Moore, 2014; Moore and Williams, 2015). These considerations on the genetic basis of evolution are not new, and have been studied for several decades (e.g., Wagner and Altenberg, 1996). Forward and reverse genetics have been highly successful in elucidating the functions of single genes or mutations for a particular trait (Nagy et al., 2003). These techniques, however, are limiting when studying the complexity of molecular interaction networks underlying a phenotype, or the molecular mechanisms of the evolution of complex traits.

The developments in genomics technology have been a major boost for our ability to study genetic complexities of phenotypic traits and their evolution (Stapley et al., 2010). Combining these techniques with the classic genetics approaches enables us to assess the functionality of genetic variation for phenotypic traits (Storz and Wheat, 2010). The first genomic studies on several model organisms emphasized that evolutionary adaptations, even for specific environmental conditions, generally govern many genes or loci, as well as the dynamic regulation of gene expression patterns (Gasch et al., 2000; Fay et al., 2004; Pedra et al., 2004). In the following decade, many studies used genomics to identify genes and proteins that were contributing to particular traits and ecological interactions. Initially, the costs and time required for sequencing a single genome were still highly restrictive. Next Generation Sequencing, however, has made it possible to sequence the genomes of many more species, and many more individuals per species. This is a formidable resource to study evolution, as it allows us, for the first time, to map the changes across the whole genome during evolution.

Genomics technology has huge potential to improve our insights into evolutionary processes. Comparative approaches have been applied to map the changes in the genome sequences or gene interaction networks at long evolutionary timescales (Drosophila 12 Genomes Consortium et al., 2007; Nowick et al., 2009; Jones et al., 2012). Experimental selection or experimental evolution approaches, followed by either transcriptomics or genome sequencing, have been used to map evolutionary changes at much shorter time scales (Hunt et al., 2010; Turner et al., 2011; Wertheim et al., 2011; Tenaillon et al., 2012; Linnen et al., 2013; Jalvingh et al., 2014). These studies showed, for example, how gene duplications, mutations, and strong sequence divergence in a small subsets of genes can have a profound impact on the transcriptional activity of large gene interaction networks and multiple phenotypic traits.

In this perspective, I present recent findings and developments on the genomic basis of evolution, using evolving immunity as a case study. Immunity is a trait that evolves rapidly, making it amenable to study the genomic basis of evolutionary processes (Obbard et al., 2009; Sironi et al., 2015). Moreover, molecular networks in immunity have been fairly well characterized due to their importance for human health (Schadt, 2009; Lazzaro and Schneider, 2014; Zak et al., 2014). Firstly, I very briefly summarize our current understanding on the molecular networks of innate immune responses and the selection processes that act on immune responses. Then, I describe the genomic changes, associated with the gain, the loss and the modulation of particular aspects of immune responses in *Drosophila*. Finally, I propose future directions to study the genetic architecture of complex traits and evolutionary processes.

## Immune Responses

The immune system consists of a combination of physiological processes that act jointly in the defense against pathogens and parasites. Innate immunity is an ancient trait that can be found in all multi-cellular organisms, while vertebrates also possess acquired immunity. Both immune systems combine cellular and humoral components: the cellular component comprises specialized cells that provide a protective function. This includes, classes of blood cells for phagocytosis of microbes, encapsulation of larger foreign bodies or recognition of antigens (in acquired immunity), and the lining of the gut with epithelial cells that form a physical barrier and can secrete defensive compounds. The humoral component consists of the release of extracellular factors that combat the invading pathogens, often from specialized tissues or cell populations. This includes the release of antimicrobial peptides (AMPs) by the liver (or the fatbody in invertebrates) and gut-epithelial cells, reactive oxygen species in phagocytic and epithelial cells, and antibodies from white blood cells (in acquired immunity; Lemaitre and Hoffmann, 2007; Buchmann, 2014).

**FIGURE 1:**
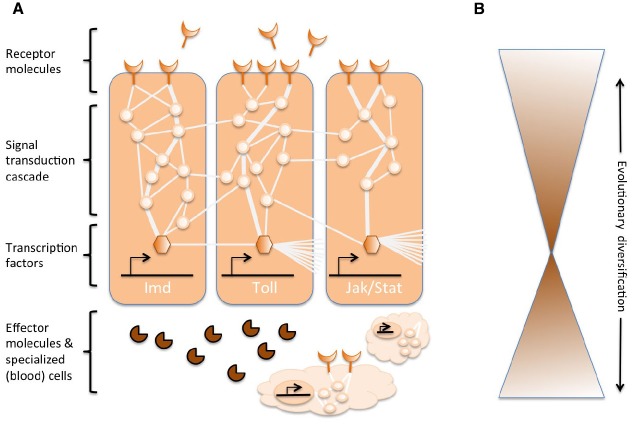
**Schematic representation of the genetic networks in immunity. (A)** Several interconnected networks coordinate the responses to an immune challenge. These networks consist of proteins (represented by circles) that interact with each in a signal transduction cascade to regulate the expression of transcription factors (represented by hexagonals). The activation of the core signal transduction pathways (e.g., IMD, Toll, or Jak/Stat, indicated by thick lines among proteins) results in the production of effector molecules, such as antimicrobial peptides (represented by pie-shaped symbols) and the proliferation and differentiation of specialized (blood) cells (cloud-shaped figures). Extracellular and membrane-bound receptor molecules (moon-shaped figures) induce the pathways. The activity can be further modulated by many other proteins that interact with the pathways and cross-talk with other pathways and genetic networks (indicated by the thin lines among proteins). **(B)** The central components of the genetic networks in immunity, e.g., the transcription factors and the proteins in direct contact with these transcription factors, are often strongly conserved across phyla. Evolutionary diversification is found more extensively toward the peripheries of the networks.

Complex networks of molecular interactions coordinate the immune responses (Figure 1A). The same pathways are central to immune responses from invertebrates to vertebrates, implying strong conservation of the core elements of molecular networks in immunity (Silverman and Maniatis, 2001; Evans et al., 2003; Buchmann, 2014). A variety of receptor molecules can recognize pathogens or parasites, for example, based on pathogen-associated molecular patterns (e.g., lipopolysaccharides specific to bacterial membranes). Once receptors are activated, they induce specific signal transduction pathways, such as the Toll, Imd, and Jak/Stat pathways (reviewed in Lemaitre and Hoffmann, 2007; Buchmann, 2014). These pathways consist of proteases, kinases, cytokines, and other proteins that eventually activate transcription factors and co-factors. Induction of these transcription factors results in production of humoral effector molecules (e.g., AMPs) and it can induce the proliferation and differentiation of cells involved in immunity. The production of different classes of blood cells is a prominent aspect of the cellular component of the immune response, both in innate and acquired immunity. In these blood cells, signal transduction cascades are also regulated to induce cell properties and proteins that effectuate the clearance of the parasite. To regulate the strength, specificity, timing and duration of immune responses, the molecular networks are modulated by cytokines, proteases, and cross-talk with other signaling pathways (Liew et al., 2005; Aggarwal and Silverman, 2008). This also includes diverse post-transcriptional regulatory networks (Ivanov and Anderson, 2013; Carpenter et al., 2014).

The drivers of the evolutionary changes in the immune responses are the combined effects of the high fitness costs of infection on the hosts, the costs of immunity, the rich diversity of pathogens and parasites that threaten the hosts, and the dynamic co-evolutionary arms races between hosts and pathogens (Schmid-Hempel, 2003). Any or all of these aspects can operate in the local environment of the host, and lead to strong selection pressures. The type of selection, however, varies from directional to purifying to balancing, depending on the costs and benefits that the host population experiences in its local environment. For example, a highly virulent pathogen invading a local community may cause a selective sweep or directional selection for particular resistance alleles, as only the hosts with these alleles may contribute to the next generations. Alternatively, a diverse or co-evolving community of pathogens may drive frequency-dependent or balancing selection, favoring the maintenance of genetic variation. Evolution of the immune responses reflect both these co-evolutionary dynamics with the parasite, and the physiological and ecological costs of the immune system (Kraaijeveld et al., 2002; Rolff and Siva-Jothy, 2003; Schmid-Hempel, 2005; Lazzaro and Little, 2009).

## Genomic Basis of Evolutionary Change in *Drosophila* Immunity

While the central machinery of immune responses is strongly conserved, several components of the extended molecular networks can evolve rapidly or diversify (Figure 1B). In *Drosophila*, rapid evolutionary change has been reported for the receptors and the effectors of immune response (Sackton et al., 2007; Obbard et al., 2009; Salazar-Jaramillo et al., 2014). These molecules operate at the interface between the host and the pathogen, and are therefore crucial for the recognition by the host of an invading organism, and to mediate the targeting and antagonistic effects of the immune response on the pathogen. At the same time, the parasite is under selection to go undetected, to avoid or mitigate the antagonistic effects of the immune response. Therefore, Red Queen dynamics are expected for these molecules at the interface, and those modulating the immune responses. Each party is trying to gain the upper hand in the antagonistic arms race, reciprocally driving alterations in the genetic networks of the parties. The diversification in receptor, modulator and effector molecules is mostly accomplished by gene duplications and rapid sequence changes (Drosophila 12 Genomes Consortium et al., 2007; Sackton et al., 2007; Salazar-Jaramillo et al., 2014).

We have been studying the immune response of *Drosophila* against parasitoid wasps as a model system to understand the genomic basis of evolutionary processes. *Drosophila* larvae are host to a variety of parasitoid species that lay an egg in these larvae (Fleury et al., 2009). Once the parasitoid egg hatches (∼2–4 days after parasitoid attack, depending on parasitoid species, and temperature), the parasitoid larva starts feeding on the host and kills it. Some species of *Drosophila* have a defense mechanism against parasitoids through an innate immune response, called melanotic encapsulation. This immune response consists of cellular and humoral components that act jointly to sequester and kill the parasitoid egg. Parasitoid attack triggers immune signal transduction pathways that induce (i) the proliferation and differentiation of two classes of hemocytes (i.e., insect blood cells) that adhere to the parasitoid egg and to each other, and (ii) the deposition of melanin on the parasitoid egg and the cellular capsule around the parasitoid egg (Lemaitre and Hoffmann, 2007). The host has to complete the full encapsulation and melanization before the parasitoid egg hatches to survive the parasitoid infestation.

Prior to the genomics era, several genes had been identified that were involved in the immune response against parasitoid wasps. The Toll and Jak/Stat pathways had been identified as central components of the hemocyte proliferation and differentiation, and the prophenoloxidase pathway for melanization (reviewed in Brennan and Anderson, 2004). Two microarray studies were then conducted to identify additional genes potentially involved in the melanotic encapsulation after parasitoid attack. This approach highlighted many additional genes that had not been previously associated to the immune response against parasitoid, and revealed their timing of action (Wertheim et al., 2005; Schlenke et al., 2007). The studies revealed several coordinated and functionally coherent clusters of genes that were temporarily up- or down-regulated during part of the immune response (Wertheim et al., 2005). Interestingly, it was shown that the virulence mechanisms of two parasitoid species differed in how they interfered in the genetic network of the hosts responses: one species eliminated the initial activation of the whole network, while another species targeted the final step in the cascade (Schlenke et al., 2007).

Species of *Drosophila* differ largely in immunity against parasitoids. Some species are completely susceptible to parasitoids and this was reported as an immune deficiency (Eslin and Doury, 2006). Closer inspection, however, revealed that parasitoid resistance is not commonly shared among all *Drosophila* species, but is restricted to a few clades. We showed that in one of those clades, the *melanogaster* subgroup, the evolutionary gain of parasitoid resistance was associated with the gain of a new type of blood cell, the lamellocytes, that is also restricted to the same clade (Salazar-Jaramillo et al., 2014). Some *Drosophila* species outside the melanogaster subgroup can also encapsulate parasitoid eggs, but they appear to have evolved different types of blood cell for the encapsulation response (Havard et al., 2012; Márkus et al., 2015). The immune response against parasitoids has evolved independently in various insect taxa, often with slightly different mechanisms and types of blood cells (Lavine and Strand, 2002). Thus, the evolution of the innate immune system includes the addition of new components or “modules.” This raises the question how the genome changes during the acquisition of a new module.

Comparative genomics revealed that, despite the gain of a new type of blood cell in the *melanogaster* subgroup, the genes that are known for lamellocyte differentiation are largely conserved across the whole phylogeny. Also species that do not produce lamellocytes in response to parasitoid attack possess these genes that are required for lamellocyte differentiation. Moreover, these genes show little divergence or signatures of selection, while that would be expected for genes that obtained a novel function. This indicates that the existing signal transduction pathways for hemocyte differentiation are being modulated by the surrounding gene interaction network to produce a novel type of blood cell in the *melanogaster* subgroup. This co-option of the existing core hemocyte proliferation pathway is likely achieved by adding other or new components to the gene interaction network (Salazar-Jaramillo et al., 2014). We identified several novel genes that arose around the time of lamellocyte acquisition and are differentially expressed during the immune response against parasitoids, including receptor molecules and serine-type proteases (Salazar-Jaramillo et al., 2014). We hypothesize that especially the serine-type endopeptidases may play a crucial role in this expansion of the gene interaction network. A substantial number of these molecules arose at the time of lamellocyte acquisition, they are expressed at the right moment in the immune response, and they show strong signatures of positive selection (Wertheim et al., 2005; Salazar-Jaramillo et al., 2014).

Also within a single species, *D. melanogaster*, immune responses show large genetic variation. Field populations collected from across Europe show substantial differences in the ability to successfully encapsulate parasitoid eggs (Kraaijeveld and van Alphen, 1995; Kraaijeveld and Godfray, 1999; Gerritsma et al., 2013). Apparently, the costs and benefits of a strong immune defense differ geographically, leading to modulation and differentiation of co-adapted genetic networks. This was also reflected in the hemocytic response after parasitoid attack. The field lines varied considerably in the absolute and relative numbers of the different hemocytes they produced in response to parasitoid attack, even among the lines that were highly successful in encapsulation (Gerritsma et al., 2013). This re-emphasizes that the genetic background of a population and the combined local selection pressures lead to alternative evolutionary responses. Comparing the genomes of resistant and susceptible individuals from several populations may reveal the adaptive variation in the genetic architecture of this trait.

To map the changes in the genome during the evolution of increased resistance, we conducted experimental evolution for parasitoid resistance. In the laboratory we exposed a large outbred population to parasitoids. Only the larvae that succeeded in surviving parasitoid attack were allowed to contribute to the next generation. With this approach, we increased the level of resistance from 20 to ∼50% of the larvae surviving parasitoid attack after only five generations of selection. When we measured changes in gene expression in the selected populations, compared to the gene expression in the control lines, even before parasitoid attack, we found several hundreds of genes that were slightly differentially regulated (Wertheim et al., 2011). The changes involved mostly genes that were not differentially expressed during the immune response, indicating that the evolutionary changes did not pre-activate the immune response in anticipation of parasitoid attack, but it modulated canonical developmental pathway, which (also) lead to an increase in its defensive abilities. We repeated this experiment, and then sequenced the genomes of the selected and control populations. In the genomes of the lines that evolved increased resistance, we found signatures of selection on multiple narrowly defined regions of the genome (Jalvingh et al., 2014). Some of these regions also overlapped with the regions that showed changed expression after selection for increased resistance (Wertheim et al., 2011; Jalvingh et al., 2014). Thus, a fast and strong selective sweep on a complex trait as immunity can still affect multiple, but highly localized, genomic regions.

## Future Challenges

How are we going to reconcile the long-term evolutionary changes, such as acquisition of new genes in gene interaction networks, and the short-term evolutionary changes, such as sequence variants that can be swept through a population? The key to this is to (i) reconstruct the gene interaction network underlying complex traits, and (ii) characterize the role of genetic variation within these networks. Genetic networks can expand with new genes through, e.g., duplications, become interconnected with other networks or modules, and small sequence variations can modulate the activity and topology of the networks. If we can decompose the genetic networks, and assess the role of genetic variants in a network context, this will eventually allow us to determine how genetic variation is translated into phenotypic variation. This will also improve our understanding of the molecular basis of complex human diseases and the evolution of innate and acquired immunity (Cooper and Alder, 2006; Manolio et al., 2009; Star et al., 2011; Mackay and Moore, 2014; Sironi et al., 2015).

Systems biological approaches will be invaluable for unraveling the complex gene interaction networks. There, mathematical models are developed to describe the molecular mechanisms underlying a trait and to predict the dynamics of groups of interacting components of the network. The models are based on molecular genetics and genomics data. At present, systems biology is mostly applied to specific traits in unicellular organisms, and this is considered the limit to what can be achieved (Papp et al., 2011). It is probable, however, that this model may not be representative for evolution in sexually reproducing multicellular organisms. Rather than waiting for the simplified models to accurately reflect small sub-networks, we need to develop and refine our methods to utilize and quantify the emergent properties from the vast amount of genomics data. We can infer gene interaction networks from protein–protein or transcript correlation or co-expression matrices (Shannon et al., 2003; Langfelder and Horvath, 2008), and merge these with natural variant analyses (Nuzhdin et al., 2012). We should further develop these methods and alternative approaches to fully exploit our measurements on genomics data, and to convert these quantitative measurements into network analyses. While genomics data in itself is not going to provide the full answer to what determines the adaptive capacity of life, it allows us to quantify and observe what happens at the molecular level during evolution. When we combine and integrate this with the environmental heterogeneity as driver of adaptations, we may be able to reveal the complex molecular mechanisms of adaptation and evolution.

### Conflict of Interest Statement

The author declares that the research was conducted in the absence of any commercial or financial relationships that could be construed as a potential conflict of interest.
